# Image Analysis Reveals Microstructural and Volumetric Differences in Glioblastoma Patients with and without Preoperative Seizures

**DOI:** 10.3390/cancers12040994

**Published:** 2020-04-17

**Authors:** Stefanie Bette, Melanie Barz, Huong Ly Nham, Thomas Huber, Maria Berndt, Arthur Sales, Friederike Schmidt-Graf, Hanno S. Meyer, Yu-Mi Ryang, Bernhard Meyer, Claus Zimmer, Jan S. Kirschke, Benedikt Wiestler, Jens Gempt

**Affiliations:** 1 Department of Neuroradiology, Klinikum rechts der Isar, Technical University of Munich, Ismaninger Str. 22, 81675 Munich, Germany; stefanie.bette@uk-augsburg.de (S.B.); huonglynham@yahoo.de (H.L.N.); maria.berndt@tum.de (M.B.); claus.zimmer@tum.de (C.Z.); jan.kirschke@tum.de (J.S.K.); b.wiestler@tum.de (B.W.); 2 Department of Diagnostic and Interventional Radiology, Universitätsklinikum Augsburg, Stenglinstr. 2, 85156 Augsburg, Germany; 3 Department of Neurosurgery, Klinikum rechts der Isar, Technical University of Munich, Ismaninger Str. 22, 81675 Munich, Germany; melanie.barz@tum.de (M.B.); arthurhenrique2@yahoo.com.br (A.S.); hanno.meyer@tum.de (H.S.M.); yu-mi.ryang@helios-gesundheit.de (Y.-M.R.); bernhard.meyer@tum.de (B.M.); 4 Department of Clinical Radiology and Nuclear Medicine, Medical Faculty Mannheim, University of Heidelberg, Theodor-Kutzer-Ufer 1–3, 68167 Mannheim, Germany; Thomas.Huber@medma.uni-heidelberg.de; 5 Department of Neurology, Klinikum rechts der Isar, Technical University of Munich, Ismaninger Str. 22, 81675 Munich, Germany; f.schmidt-graf@tum.de; 6 Department of Neurosurgery, HELIOS Klinikum Berlin-Buch, Schwanebecker Chaussee 50, 13125 Berlin, Germany

**Keywords:** seizures, glioblastoma, diffusion-tensor-imaging

## Abstract

*Purpose*: Seizures related to tumor growth are common in glioma patients, especially in low-grade glioma patients this is often the first tumor manifestation. We hypothesize that there are associations between preoperative seizures and morphologic features (e.g., tumor size, location) and histogram features in patients with glioblastoma (GB). *Methods*: Retrospectively, 160 consecutive patients with initial diagnosis and surgery of GB (WHO IV) and preoperative MRI were analyzed. Preoperative MRI sequences were co-registered (T2-FLAIR, T1-contrast, DTI) and tumors were segmented by a neuroradiologist using the software ITK-snap blinded to the clinical data. Tumor volume (FLAIR, T1-contrast) and histogram analyses of ADC- and FA-maps were recorded in the contrast enhancing tumor part (CET) and the non-enhancing peritumoral edema (FLAIR). Location was determined after co-registration of the data with an atlas. Permutation-based multiple-testing adjusted t statistics were calculated to compare imaging variables between patients with and without seizures. *Results*: Patients with seizures showed significantly smaller tumors (CET, adj. *p* = 0.029) than patients without preoperative seizures. Less seizures were observed in patients with tumor location in the right cingulate gyrus (adj. *p* = 0.048) and in the right caudate nucleus (adj. *p* = 0.009). Significant differences of histogram analyses of FA in the contrast enhancing tumor part were observed between patients with and without seizures considering also tumor location and size. *Conclusion*: Preoperative seizures in GB patients are associated with lower preoperative tumor volume. The different histogram analyses suggest that there might be microstructural differences in the contrast enhancing tumor part of patients with seizures measured by fractional anisotropy. Higher variance of GB presenting without seizures might indicate a more aggressive growth of these tumors.

## 1. Introduction

Brain tumor-related epilepsy (BTE) is a common symptom of patients with intracranial gliomas and occurs in about 50% of high-grade and up to 100% of low-grade glioma patients [1,2,3,4,5]. Many studies investigated the pathophysiological mechanisms of BTE and its risk factors [6,7]. Mutation of the isocitrate dehydrogenase 1/2 [8] (IDH1/2-) gene was shown to correlate with seizures in low-grade gliomas due to the production of D-2-hydroxyglutarate which is similar to glutamate, an excitatory neurotransmitter that initiates NMDA-receptor related pathways [1,9,10,11,12]. Many other factors are involved in the pathophysiology of BTE such as ionic changes, GABAergic pathways, hypoxia and inflammatory changes [12]. The tumor location and the type of the tumor play an important role, slowly growing tumors have a higher risk of seizures [4,12]. Seizure as first manifestation of the tumor was also shown to be associated with a better prognosis [5]. Also the peritumoral region has attracted attention in the pathophysiology of BTE [13,14,15,16]. Studies showed that high-grade glioma patients with smaller preoperative tumor size significantly more often presented with seizures, whereas for low-grade gliomas contradicting results were shown [17,18].

Diffusion tensor imaging (DTI) is routinely used in preoperative glioma imaging and provides an insight into the microstructure of tumors and brain tissue [19]. The main quantitative values assessed via DTI are the apparent diffusion coefficient (ADC) and fractional anisotropy (FA). Studies showed that not only the contrast-enhancing tumor but also the non-enhancing peritumoral region demonstrate differences in the microstructure measured especially by FA [20,21,22]. 

To our knowledge, there are no studies that assessed the relationship between preoperative seizures and FA/ADC values in glioblastoma patients. 

We therefore hypothesize that glioblastoma causing preoperative seizures differ from glioblastoma causing no preoperative seizures in size, location and microstructure measured by DTI. 

## 2. Methods

This retrospective single-center non-interventional study was approved by the local ethics committee (5625-12) at the Klinikum rechts der Isar of the Technical University of Munich, Germany and performed in accordance with the ethical standards of the 1964 Declaration of Helsinki and its later amendments [23]. 

### 2.1. Patient Population

From the local database 160 consecutive patients with surgery for a newly diagnosed glioblastoma (GB) (WHO IV) between 01/2010 and 12/2015 were included in this study. They were selected from a collective of 260 patients choosing those with completely recorded clinical data, especially concerning epilepsy data, as well as complete preoperative in-house imaging protocol included high-resolution magnetic resonance imaging (T1-weighted imaging after contrast agent, Fluid-Attenuated-Inversion-Recovery (FLAIR) images and diffusion tensor imaging (DTI)). Only patients with first diagnosis of glioblastoma in a preoperative stage, not having received any chemotherapy or radiotherapy before date of image acquisition, were selected. The occurrence of seizure as initial tumor manifestation was recorded by qualified neurosurgeons. Isocitrate-dehydrogenase 1 (IDH1)-mutation status was assessed in 109/160 patients in the local department of neuropathology via immunostaining against the R132H mutation in all patients.

### 2.2. MR Imaging

MRI scans were performed on a 3 Tesla (T) MRI scanner, either Philips Achieva, Philips Ingenia (Philips Medical Systems, The Netherlands B.V.) or Siemens Verio (Siemens Healthcare, Erlangen, Germany). All patients had FLAIR-images, high-resolution T1-weighted (w) images with and without contrast agent (MPRage, 1 mm isotropic) and Diffusion Tensor Imaging (DTI). DTI sequence either comprised 6 diffusion directions (b value 800 s/mm^2^, TR/TE 7665/55 ms, resolution 2 × 2 × 2 mm) or 15 diffusion directions (b value 800 s/mm^2^, TR/TE 10728/55 ms, resolution 2 × 2 × 2 mm) or 15 diffusion directions (b1000, TR/TE 7665/55 ms, resolution 2 × 2 × 2 mm). The contrast agent Magnograf® was administered intravenously by a standardized protocol (0.2 mL/kg, 0.5–1 mL/sec), using a MR compatible contrast medium injection system (Spectris Solaris EP, Siemens Medical, Erlangen, Germany). 

### 2.3. Image Analysis

**Figure 1:**
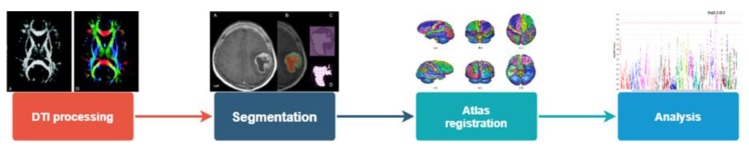
Flow chart of image analysis and data processing.

Image analysis was supervised by two neuroradiologists (BW, 7 years of experience and SB, 7 years of experience) blinded to the clinical data. Image pre-processing encompassed N4 bias-field correction and linear co-registration using the open-source ANTs packages (https://stnava.github.io/ANTs/) [24]. DTI processing was done with DiPy (https://nipy.org/dipy/) [25], including affine registration of diffusion-weighted images to the b0 image and appropriate vector rotation and non-linear estimation of the diffusion tensor. Semi-automatic segmentation of tumors in two mutually exclusive areas (contrast-enhancing and FLAIR-hyperintense tumor) was performed using a generative probabilistic model [26]. Lesion-filled T1 images were deformably registered (SyN) to the SRI24 atlas [27]. Resulting segmentations and atlas images were checked manually prior to analysis with the freely available software ITK-SNAP (www.itksnap.org) [28]. From the co-registered FA and ADC maps, first-order statistics were automatically calculated using the PyRadiomics package (https://pyradiomics.readthedocs.io/) [29] in both contrast-enhancing and FLAIR-hyperintense tumor areas. Volume information and atlas locations were collected for both areas (Figure 1). Tumor size was calculated by counting voxels (each voxel with a size of 1 × 1 × 1 mm) and shown as mm^3^. For atlas localization, the extent of the entire mass was analyzed. All scripts are available upon request from B.W.

### 2.4. Statistics

Statistical analysis including descriptive data analysis was performed using IBM SPSS Statistics version 24.0 (SPSS Inc., IBM Corp., Armonk, NY, USA), Python version 3.6 (https://www.python.org/) and R version 3.5 (https://www.r-project.org/). To compare first-order statistics in patients with and without seizures and account for multiple testing, random label permutations (with 1000 iterations) were performed as described previously [30]. Wilcoxon tests were performed for correlations of histogram analyses and tumor location (location of the tumor in a brain region that was significantly associated with seizures in this cohort vs. location of the tumor in another region), Pearson correlation analyses were performed to analyze the influence of tumor volume on histogram analyses. 

A difference with an error probability of less than 0.05 was considered as statistically significant.

## 3. Results

### 3.1. Patients’ and Tumor Characteristics

The study population comprises 160 consecutive patients (90 male, mean age 64y +/- 13.9) with initial diagnosis of a glioblastoma (WHO IV) (Table 1). 60/160 patients presented with preoperative seizures. 87/160 tumors showed infiltration of the frontal lobe, 93/160 tumors infiltrated the temporal lobe. Infiltration of the parietal lobe was shown in 57/160 cases, of the occipital lobe in 37/160 cases. 90/160 tumors showed infiltration of the insular region, 51/160 tumors of the hippocampus. The brainstem was infiltrated in 13/160 cases, the cerebellum in 4/160 cases.

### 3.2. Tumor Size and Location

Patients with seizures showed significantly smaller tumors (contrast enhancing tumor) (adj. *p* = 0.029) than patients without preoperative seizures. FLAIR volume did not significantly differ between patients with and without preoperative seizures (adj. *p* = 0.725) (Table 2). 

**Figure 2:**
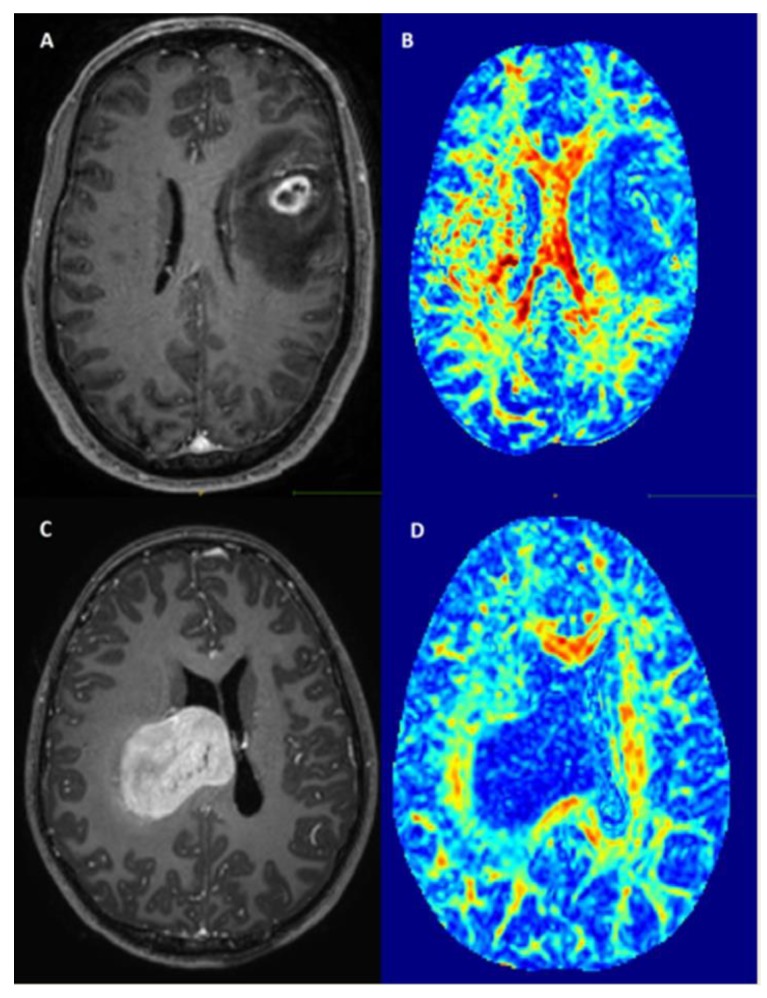
Examples of patients with (**A**) = T1-weighted imaging after contrast agent, (**B)** = Fractional anisotropy maps) and without (**C**,**D**) preoperative seizures.

Patients with tumor location in the right cingulate gyrus (adj. *p* = 0.048) and in the right caudate nucleus (adj. *p* = 0.009) showed significantly less preoperative seizures (Table 3). Patients with tumors in the limbic system (including parahippocampal and hippocampal gyrus and cingulate gyrus) also showed significantly less preoperative seizures (*p* = 0.030) All other tumor locations were not associated with the occurrence of preoperative seizures. Figure 2 shows examples of a patient with a small tumor in the left frontal lobe presenting with seizures (A,B) and a patient with a large tumor in the right cingulate gyrus presenting without preoperative seizures (C,D). 

### 3.3. Histogram Analyses

**Figure 3:**
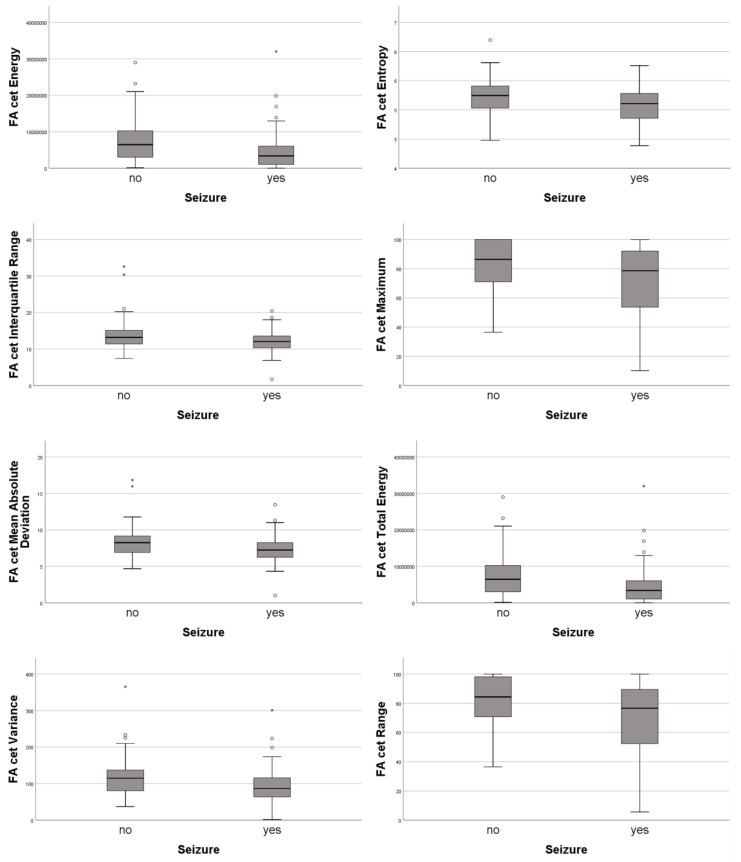
Boxplots for histogram analyses.

Histogram analyses of fractional anisotropy (FA) in the contrast enhancing tumor part significantly differed between patients with and without preoperative seizures. The following features showed significant differences: Energy (adj. *p* = 0.017), Entropy (adj. *p* = 0.043), Interquartile Range (adj. *p* = 0.013), Maximum (adj. *p* = 0.043), Mean Absolute Deviation (adj. *p* = 0.017), Range (adj. *p* = 0.025), Total Energy (adj. *p* = 0.017), Variance (adj. *p* = 0.039) (Figure 3). All features were significantly smaller in patients presenting with seizures.

No significant differences were observed between histogram analyses of ADC in the contrast enhancing tumor part and of FA and ADC in the FLAIR-hyperintense part (Table S1). 

Analyses for correlations between tumor location and FA histogram analyses showed no significant differences (Figure S1). 

Tumor size showed significant positive correlations to the following FA histogram features: Energy, Entropy, Maximum, Range und Total Energy. No significant correlations were observed between tumor size and the features Interquartile Range, Variance and Mean Absolute Deviation (Figure S1). Significant correlations were shown between the FA features that are independent of tumor size: Interquartile Range/Variance: *r* = 0.927, *p* < 0.001; Interquartile Range/Mean Absolute Deviation: *r* = 0.966, *p* < 0.001, Mean Absolute Deviation/Variance: *r* = 0.963, *p* < 0.001. 

## 4. Discussion

Glioblastoma patients with preoperative seizures show significantly smaller tumors. Tumor location in the right cingulate gyrus and in the right caudate nucleus were associated with less preoperative seizures whereas infiltration of the hippocampus and the insula did not appear to promote epileptogenesis.

Tumors with and without preoperative seizures differed in histogram analyses of FA in the contrast enhancing tumor – with smaller values in the features Interquartile Range, Mean Absolute Deviation and Variance after consideration of tumor size. As glioma patients with preoperative seizures were shown to have a better prognosis [31], this might be reflected by differences in the microstructure.

Brain tumor-related epilepsy was shown to mainly occur in low-grade glioma patients, whereas glioblastoma patients more often present with other symptoms such as neurologic deficits or headache due to the mass effect [4,5,7,17]. As seizures were shown to be associated with improved survival in high-grade glioma [31], it is of high importance to characterize the exact pathomechanisms causing seizures in glioma patients to develop new therapy strategies. Many studies investigated the pathomechanisms for brain tumor-related seizures [1,9,10,11,13,16,18,31]. There are two main hypotheses: First, the mechanical compression of surrounding brain structures by the tumor mass might cause seizures which is supported by the findings that gross-total tumor resection is associated with seizure-control [31,32]. Second, the tumor excretes epileptogenic factors such as glutamate or causes altered gene expression in the peritumoral region which results in seizures [31,33,34,35].

The main results of this study are that preoperative tumor volume of the contrast enhancing tumor (not the FLAIR-hyperintense edema) shows a significant correlation to seizures. This is in common with a previous study by Skardelly et al. that showed a tumor volume <64 cm^3^ as a main risk factor for the development of preoperative seizures [18]. Another study showed that this association was only found in high-grade gliomas, but not in low-grade gliomas where an inverse association between tumor volume and seizures was observed [17]. The mentioned study by Skardelly et al. reported a large population of 242 glioblastoma patients. More rapidly growing tumors might more often be associated with other symptoms such as hemiparesis, aphasia or headache due to the mass effect [17]. In contrast, small tumors are lacking this space-consuming effect. These results would suggest that smaller tumors might have a higher epileptogenic level than larger high-grade tumors. A possible pathomechanism might be that these smaller tumors excrete epileptogenic factors or perform changes in the peritumoral region as it was discussed in previous studies [9,33,34]. 

At this point a parallel to low grade gliomas, especially to the larger ones with a relevant surround reaction and higher level of aggressiveness and growth rate as in small ones, could be seen, that consequently also show a higher epileptogenic potential [17]. 

It remains to be seen if these small high-grade gliomas are at an early stage of growth with an early diagnosis due to seizures as their primary symptom or if they represent a slowly growing, overall less aggressive tumor type with a higher epileptogenic potential. Further studies that investigate the exact pathophysiologic mechanisms of tumor growth and seizures will have to be performed to better characterize these tumor types. 

In the present study, glioblastomas without seizures showed a preference towards the right cingulate gyrus and the right caudate nucleus. These findings are in common with a previous study by Lee JW et al. [17]. According to this study high grade gliomas presenting with neurologic symptoms instead of seizures were more likely to occur in the pericallosal region [17]. Interestingly other than previous studies, we found no significant associations between location in the left hemisphere and infiltration of the hippocampus was associated with preoperative seizure as shown in previous studies [18,31,32]. Main explanation for this finding might be that the cited previous studies mainly assessed low-grade gliomas whereas this study only investigated glioblastoma patients only. 

Histogram analyses of FA in the contrast enhancing tumor significantly differed between patients with and without seizures suggesting that there are microstructural differences in this tumor area. 

Previous studies analyzed FA values in the CE tumor and showed that GB have higher FA values than brain metastases [36,37,38,39]. Higher FA values in the CE tumor were explained as an overproduction of extracellular matrix by glioblastoma cells that accumulate in the CE tumor area [37,39,40,41]. These results might suggest that especially the contrast enhancing tumor area attracts attention for further studies concerning the pathophysiology of preoperative seizures. 

Tumors presenting with seizures showed significantly lower values in the features Energy, Entropy, Maximum, Range, Total Energy, Interquartile Range, Mean Absolute Deviation and Variance. The features Energy, Entropy, Maximum, Range and Total Energy were also associated with tumor size, therefore this difference might be explained as tumors presenting with seizures are significantly smaller. Interquartile Range, Mean Absolute Deviation and Variance however, were independently smaller in tumors presenting with seizures. These three FA measures-Interquartile Range, Mean Absolute Deviation and Variance–are all associated with FA variability and are highly correlated with each other. 

These results might suggest that tumors presenting with seizures are more homogenous and show a growth similar to low-grade tumors (that also present with seizures more often than high-grade tumors). On the other hand, tumors presenting without seizures might show a more inhomogenous/aggressive growth.

Main limitation of this study is its retrospective design. The semiautomatic segmentation is a reliable tool for measurement of tumor volume but also associated with precision errors [42]. However, by now this might be considered state of the art and both, the volumetric measurements and the qualitative data analysis, were performed blinded to the clinical data to reduce this bias. Another limitation is the fact that the results were not validated in an independent cohort. Therefore, further studies are necessary to confirm the results of this study. 

## 5. Conclusions

In glioblastoma patients, preoperative seizures were associated with significantly smaller contrast enhancing tumor volumes. Tumor location in the right cingulate gyrus and caudate nucleus were associated with less preoperative seizures. Significant differences in histogram analyses of FA in the contrast enhancing tumor part were observed suggesting that there are microstructural differences between these tumors. As glioblastomas with preoperative seizures are associated with an improved survival it is important to investigate the exact pathomechanisms causing brain-tumor related epilepsy. 

## Figures and Tables

**Table 1:** Baseline patient and tumor characteristics.

Age	64 Years (+/-13.9)
Sex, male	90/160
Preoperative seizure	60/160
Tumor infiltration of	
- frontal lobe	87/160
- temporal lobe	93/160
- parietal lobe	57/160
- occipital lobe	37/160
- insula	90/160
- hippocampus	51/160
- cerebellum	4/160
- brainstem	13/160
Hemisphere	
- right	74/160
- left	64/160
- both	22/160
IDH1-wildtype	108/109

Normally distributed variables shown as mean +/- standard deviation.

**Table 2:** Tumor size.

MR Sequence	Seizure	No Seizure	adj. *p*-Value
FLAIR	48884.4 mm^3^ (31830.9–97074.7)	85391.7 mm^3^ (54260.2–124244.1)	0.725
CET*	8434.9 mm^3^ (3604.9–17662.9)	14805.7 mm^3^ (8196.8–26347.5)	0.029

Data shown as median (interquartile range); CET: contrast enhancing tumor, FLAIR: fluid-attenuated inversion recovery; * *p* < 0.05.

**Table 3:** Tumor location and seizures.

Location	Seizure (*n* = 60)	No Seizure (*n* = 100)	Odds Ratio	Perm. *p*-Value
Left superior frontal gyrus	17	38	0.645	0.857
Right superior frontal gyrus	19	43	0.614	0.140
Left middle frontal gyrus	16	29	0.890	1
Right middle frontal gyrus	15	41	0.480	0.172
Left inferior frontal gyrus	16	27	0.983	1
Right inferior frontal gyrus	13	37	0.471	0.248
Left precentral gyrus	19	30	1.081	1
Right precentral gyrus	18	50	0.429	0.091
Left middle orbitofrontal gyrus	9	20	0.706	1
Right middle orbitofrontal gyrus	4	25	0.214	0.064
Left lateral orbitofrontal gyrus	9	19	0.752	1
Right lateral orbitofrontal gyrus	6	27	0.300	0.214
Left gyrus rectus	2	12	0.253	1
Right gyrus rectus	4	11	0.578	0.783
Left postcentral gyrus	15	21	1.254	0.999
Right postcentral gyrus	16	46	0.427	0.200
Left superior parietal gyrus	9	14	1.084	1
Right superior parietal gyrus	15	29	0.816	1
Left supramarginal gyrus	9	12	1.294	1
Right supramarginal gyrus	10	30	0.467	0.072
Left angular gyrus	14	16	1.598	0.991
Right angular gyrus	12	26	0.712	0.935
Left precuneus	9	13	1.181	1
Right precuneus	11	26	0.639	1
Left superior occipital gyrus	6	7	1.476	1
Right superior occipital gyrus	8	21	0.579	0.873
Left middle occipital gyrus	9	9	1.784	0.449
Right middle occipital gyrus	10	21	0.752	1
Left inferior occipital gyrus	7	10	1.189	1
Right inferior occipital gyrus	8	10	1.385	1
Left cuneus	8	8	1.769	0.995
Right cuneus	7	13	0.884	1
Left superior temporal gyrus	24	31	1.484	0.943
Right superior temporal gyrus	18	46	0.503	0.266
Left middle temporal gyrus	21	27	1.456	0.999
Right middle temporal gyrus	14	39	0.476	0.125
Left inferior temporal gyrus	20	26	1.423	0.998
Right inferior temporal gyrus	15	26	0.949	1
Left parahippocampal gyrus	19	27	1.253	1
Right parahippocampal gyrus	14	31	0.677	0968
Left lingual gyrus	11	19	0.957	1
Right lingual gyrus	12	25	0.750	0.995
Left fusiform gyrus	17	24	1.252	1
Right fusiform gyrus	13	30	0.645	0.999
Left insular cortex	18	32	0.911	1
Right insular cortex	18	46	0.503	0.266
Left cingulate gyrus	18	42	0.592	0.877
*Right cingulate gyrus*	*19*	*49*	*0.482*	*0.048*
Left caudate	19	35	0.861	1
*Right caudate*	*14*	*45*	*0.372*	*0.009*
Left putamen	20	36	0.889	1
Right putamen	19	49	0.482	0.187
Left hippocampus	18	27	1.159	1
Right hippocampus	15	36	0.593	0.476
cerebellum	9	15	1.000	1
brainstem	12	29	0.612	1

## Supplementary Materials

The following are available online at https://www.mdpi.com/2072-6694/12/4/994/s1, Figure S1: Box plots for FA first order features in tumors in epilepsy location/different location, Table S1: First order features.

## Abbreviations

ADC	**Apparent diffusion coefficient**
BTE	Brain tumor-related epilepsy
FA	Fractional anisotropy
FLAIR	Fluid-attenuated inversion recovery
GB	Glioblastoma
MPRage	Magnetization prepared rapid gradient echo
