# Supplementary material for: Image Analysis Reveals Microstructural and Volumetric Differences in Glioblastoma Patients with and without Preoperative Seizures
Source: Cancers (Basel). 2020 Apr 17;12(4):994. doi: 10.3390/cancers12040994 (PMC7226080; doi:10.3390/cancers12040994)
Supplement: Supplementary file 1 [file cancers-12-00994-s001.pdf]

Supplementary Materials

Image Analysis Reveals Microstructural and Volumetric Differences in Glioblastoma Patients with and without Preoperative Seizures

Stefanie Bette, Melanie Barz, Huong Ly Nham, Thomas Huber, Maria Berndt, Arthur Sales, Friederike Schmidt-Graf, Hanno S. Meyer, Yu-Mi Ryang, Bernhard Meyer, Claus Zimmer, Jan S. Kirschke, Benedikt Wiestler and Jens Gempt

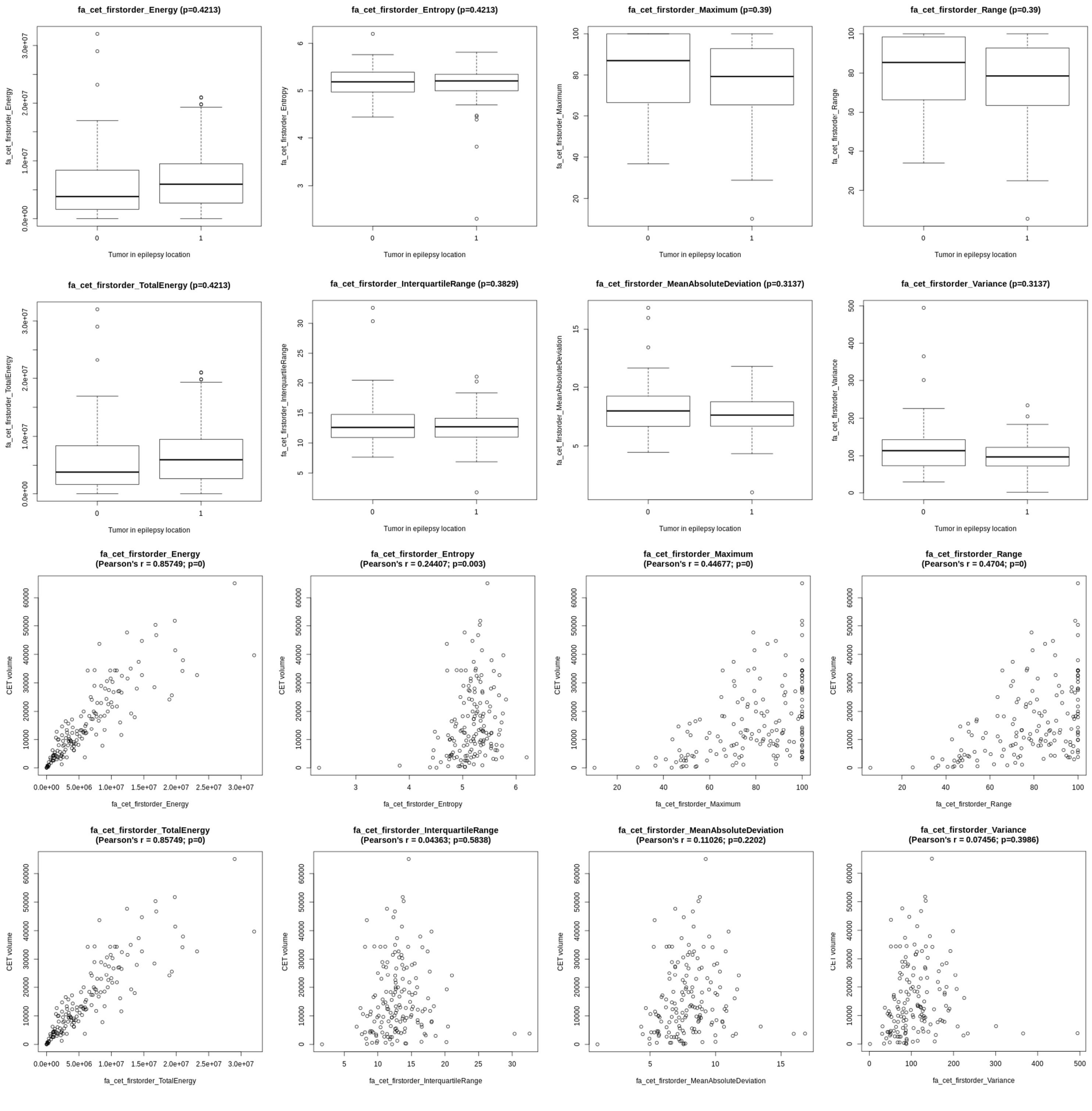

Figure S1. Box plots for FA first order features in tumors in epilepsy location / different location. Scatter plots for FA first order features and tumor volume of the contrast-enhancing tumor (CET).

Table S1. First order features.

Feature perm.	*p*-Value
fa_cet_firstorder_10Percentile	1
fa_cet_firstorder_90Percentile	0.192807193
fa_cet_firstorder_Energy	0.016983017*
fa_cet_firstorder_Entropy	0.042957043*
fa_cet_firstorder_InterquartileRange	0.012987013*
fa_cet_firstorder_Kurtosis	1
fa_cet_firstorder_Maximum	0.042957043*
fa_cet_firstorder_MeanAbsoluteDeviation	0.016983017*
fa_cet_firstorder_Mean	0.959040959
fa_cet_firstorder_Median	1
fa_cet_firstorder_Minimum	0.862137862
fa_cet_firstorder_Range	0.024975025*
fa_cet_firstorder_RobustMeanAbsoluteDeviation	0.051948052
fa_cet_firstorder_RootMeanSquared	0.71028971
fa_cet_firstorder_Skewness	0.68031968
fa_cet_firstorder_TotalEnergy	0.016983017*
fa_cet_firstorder_Uniformity	0.882117882
fa_cet_firstorder_Variance	0.038961039*
adc_cet_firstorder_10Percentile	1
adc_cet_firstorder_90Percentile	1
adc_cet_firstorder_Energy	0.999000999
adc_cet_firstorder_Entropy	1
adc_cet_firstorder_InterquartileRange	1
adc_cet_firstorder_Kurtosis	0.992007992
adc_cet_firstorder_Maximum	0.996003996
adc_cet_firstorder_MeanAbsoluteDeviation	1
adc_cet_firstorder_Mean	1
adc_cet_firstorder_Median	1
adc_cet_firstorder_Minimum	1
adc_cet_firstorder_Range	0.974025974
adc_cet_firstorder_RobustMeanAbsoluteDeviation	1
adc_cet_firstorder_RootMeanSquared	1
adc_cet_firstorder_Skewness	1
adc_cet_firstorder_TotalEnergy	0.999000999
adc_cet_firstorder_Uniformity	1
adc_cet_firstorder_Variance	1
fa_flair_firstorder_10Percentile	1
fa_flair_firstorder_90Percentile	1
fa_flair_firstorder_Energy	0.984015984
fa_flair_firstorder_Entropy	1
fa_flair_firstorder_InterquartileRange	1
fa_flair_firstorder_Kurtosis	1
fa_flair_firstorder_Maximum	1
fa_flair_firstorder_MeanAbsoluteDeviation	1
fa_flair_firstorder_Mean	1
fa_flair_firstorder_Median	1
fa_flair_firstorder_Minimum	0.703296703
fa_flair_firstorder_Range	0.999000999
fa_flair_firstorder_RobustMeanAbsoluteDeviation	1
fa_flair_firstorder_RootMeanSquared	1
fa_flair_firstorder_Skewness	1
fa_flair_firstorder_TotalEnergy	0.984015984
fa_flair_firstorder_Uniformity	1
fa_flair_firstorder_Variance	1
adc_flair_firstorder_10Percentile	1

adc_flair_firstorder_90Percentile	1
adc_flair_firstorder_Energy	0.432567433
adc_flair_firstorder_Entropy	1
adc_flair_firstorder_InterquartileRange	1
adc_flair_firstorder_Kurtosis	1
adc_flair_firstorder_Maximum	1
adc_flair_firstorder_MeanAbsoluteDeviation	1
adc_flair_firstorder_Mean	1
adc_flair_firstorder_Median	1
adc_flair_firstorder_Minimum	0.999000999
adc_flair_firstorder_Range	1
adc_flair_firstorder_RobustMeanAbsoluteDeviation	1
adc_flair_firstorder_RootMeanSquared	1
adc_flair_firstorder_Skewness	1
adc_flair_firstorder_TotalEnergy	0.432567433
adc_flair_firstorder_Uniformity	0.995004995
adc_flair_firstorder_Variance	1
cet_volume	0.028971029*
flair_volume	0.725274725

FA: fractional anisotropy, ADC: apparent diffusion coefficient, CET: contrast enhancing tumor, FLAIR: fluid-attenuated inversion recovery. * $p < 0.05$.

© 2020 by the authors. Licensee MDPI, Basel, Switzerland. This article is an open access article distributed under the terms and conditions of the Creative Commons Attribution (CC BY) license (http://creativecommons.org/licenses/by/4.0/).
